# Medical Opinion and Movement

**Published:** 1910-05-07

**Authors:** 


### Medical Opinion and Movement.

### Anæsthetics and the Leucocytes.

At a meeting of the *Académie de Medecine* Dr. Achard read an interesting paper on the effect of anæsthetics upon the leucocytes of the blood. According to his observations both ether and chloroform administered in the ordinary way have a paralysing effect upon the activity of these white corpuscles, and in cases in which the administration has been prolonged to an hour or more their activity is almost completely abolished. This paralysing effect is only transitory and passes off in the course of 24 hours, but it persists for some time after the patient has recovered from the anæsthetic. The author suggests that this phenomenon indicates that anæsthetics exert their influence upon the whole organism, even upon those cells which are independent of direct influence from the nervous system, and harmonises with the fact that in spite of the rapidity of their action anæsthetics are only eliminated slowly from the system. He suggests further that this paralysing effect of anæsthetics upon the leucocytes may in some measure account for certain post-operative sequelæ, such as infection of the respiratory or digestive tracts owing to the consequent diminished resisting power of the organism.

### A New Method of Excluding the Pylorus.

In cases of cancer of the pylorus gastro-enterostomy is performed to relieve the obstruction and also to give rest to the affected part and relieve it from irritation caused by the passage of food. The operation does not, however, altogether effect this latter desideratum, as it has been shown by radioscopic examination that a certain amount of food, varying in different cases, continues to pass by the natural channel. The classic method to exclude the pylorus entirely consists in a vertical section of the stomach and occlusion of the divided ends, but this necessarily prolongs the operation, a matter often of serious importance in such cases. Professor G. Parlavecchio, of Palermo, proposes a much simpler method of procedure, which he has carried out successfully on dogs. It consists in ligature of the whole stomach at a sufficient distance from the pylorus. A curved pair of forceps carrying a thick silk thread is passed through the gastro-colic omentum at the level of the greater curvature, carried up behind the stomach, and then passed through the gastro-hepatic omentum from behind forwards. In this way the thread is made to surround the stomach, and it is then tied sufficiently tight to obstruct the passage, but not so as to strangle the gastric walls. With just such sufficient tension, according to this author, a circular cicatrix is formed, the muscular layers atrophy and are absorbed, and are replaced by a resistant fibrous band. The healthy part of the stomach is, of course, anastomosed to the jejunum by the ordinary method of gastro-enterostomy. It remains for future experience to show whether the method is safe and at the same time efficacious.

### Atheroma and Diet.

Among the many supposed causes of atheroma of the vascular system an excessive meat diet has been considered to occupy an important position. In order to elucidate further the pathology of this disease a good deal of research work has been carried out upon animals in the endeavour to produce the disease experimentally by various methods. Gilbert and Sion claimed to have produced atheroma of the aorta in rabbits by the injection of cultures and bacterial toxines, and Josné noted similar results after injections of 1 in 1,000 adrenalin solution. More recently the same effects have been reported from injections of various substances, including nicotine. But at the same time these results have been a good deal discounted by the discovery that rabbits are liable to develop the disease spontaneously. Dr. Weinberg has taken up the subject from this point of view, and has examined a large number of animals of various kinds. His results are extremely interesting both on account of the frequency of the occurrence of the disease spontaneously and also on account of the bearing that his results have on the question of the relation of the disease to meat diet. He found 37 cases of atheroma in 562 rabbits, and the subsequent examination of a number of batches of rabbits, amounting to 420, showed a percentage of atheroma cases varying from 4 to 19 per cent. Among 500 guinea-pigs no atheromatous lesions were found. Among 1,511 horses atheroma of the aorta with calcareous deposits similar to that found in rabbits occurred 115 times, or in 7.6 per cent. Forty-four similar lesions were found in 1,047 dogs examined. On the other hand, no such lesions were found among 344 cats, 926 sewer rats, 51 urubu (a bird of prey), and 30 dog-fish. The disease appears to be present in considerable proportions among the herbivorous animals and entirely absent among the carnivorous, whereas in the omnivorous animals, such as the dog, it is present in a much less degree. On the other hand, its frequent occurrence in rabbits entirely vitiates the experimental results with these animals, as already shown by Dr. Kalamkarov.

### Pathology of Perforating Ulcer of the Foot.

It is generally recognised that perforating ulcer of the foot is an affection involving, as the primary seat of the trouble, the skin and subcutaneous tissue, the bones and articulations only becoming implicated in the later stages as the morbid process invades the deeper tissues. According, however, to the observations of Dr. R. Levy, of Breslau, this view is incorrect and altogether misleading. His observations are based upon 13 cases of typical perforating ulcers, in all of which he was able to trace the primary source of the trouble to an affection of the underlying bones or articulations. One case was that of a man, aged 40, suffering from syringomyelia and with an ulcer at the level of the metatarso-phalangeal articulation of the third toe. Microscopic examination showed it to be of a tuberculous nature, and radioscopic examination did not disclose any osseous lesion; but the case is instructive as showing the fallacy of supposing such an ulcer necessarily of a trophic nature in association with a disease of the nervous system. In all the other cases radioscopy showed the presence of a primary morbid focus in the bones or joints of the foot. The author emphasises the importance of radioscopy, as exploration with a probe may not disclose any connection between the ulcer and the deeper primary focus. According to the author, on questioning the patient it will generally be found that the affected part was swollen and hyperæmic for some time before the ulcer appeared. One of the cases narrated as typical of the condition was that of a man aged 36, who had had a painful swelling of the right foot for three years, whereas the perforating ulcer had only appeared three weeks ago. Radiography showed the presence of a spontaneous double fracture of the fifth metatarsal. The importance of recognising such deeper lesions is obvious, as superficial treatment must necessarily fail under such circumstances, and the author attributes the failure of treatment hitherto of perforating ulcer to the fact that the involvement of the skeletal tissues has been generally overlooked.

### Foul Air and Bacillary Activity.

Dr. Trillat has recently reported to the Paris Academy of Medicine the result of some experiments he has carried out with the object of determining the influence of vitiated air upon the vitality of micro-organisms. He finds that, on exposing various pathogenic organisms, such as the bacillus diphtheriæ, the B. typhosus, and the B. pestis to an atmosphere of artificially befouled air, they increase and multiply much more quickly than when exposed to normal air. This effect is the more marked if the bacilli have been previously attenuated. From these experiments the author claims to show the important rôle played by impure air in the propagation of epidemics.

### Thyroid Extract for Eye Diseases.

Yet another condition, or rather set of conditions, is being treated by thyroid extract, with satisfactory results. Mr. Percy Dunn reports in the *Clinical Journal* his experience of this substance, to which he gave a trial on the strength of an article in an American medical paper. He ordered it first for a child who developed keratitis after the enucleation of the other eye for traumatic panophthalmitis. The lesion, which was syphilitic in origin, and had resisted all treatment for a fortnight, yielded at once to three-grain doses of thyroid extract. Incidentally it may be noted that Mr. Dunn regards interstitial keratitis as a nutritional effect occurring in syphilitic children, not as a manifestation of syphilis itself; he therefore does not treat it with mercury. The worst cases are always found in ill-nourished children, the mildest in those who appear well-nourished. After this initial success the author tried the same substance for rheumatic iritis, for glaucoma, and for obstinate corneal ulcer; in all these diseases rapid improvement coincided with the exhibition of the thyroid extract. If further experience confirms the almost magical powers attributed to the treatment, the task of the ophthalmic surgeon will be much lightened. The drawback to the method, according to the author, is that it is empirical; but symptoms of thyroidism are not produced, which is a much more important point. The dosage employed was uniform for both adults and children, namely, three grains three times a day.

### Factitious Albuminuria.

That palpation of the abdomen by a heavy-handed examiner may cause unnecessary pain to the examinee is well known, and is sufficient reason, apart from the muscular resistance evoked, for gentleness in such manipulations. But Schreiber in the *Deutsch. Archiv. fur Klin. Med.* describes albuminuria as a hitherto unknown sequela, and finds that he can produce this phenomenon practically at will in patients with fairly thin abdominal walls. The exact position at which he applies pressure for this purpose is at the level of the second lumbar vertebra, where the renal arteries arise from the aorta; sustained palpation of the aorta here will lead to albuminuria shortly afterwards. The patient passes his urine just before the pressure is applied, and at intervals of five minutes afterwards. Schreiber says that the urine secreted during the actual compression of the aorta is probably free from albumin, and that it is that secreted subsequently that is albuminous. There would appear to be no organic lesions of the renal tissue; the effect is thought to be due to local fall of blood-pressure. The phenomenon may last from ten minutes up to twenty-four hours. Both serum-albumin and serum-globulin are present, but no casts or blood-cells are passed. In rabbits, compression of the aorta below the renal arteries will cause albuminuria, but this effect is not produced in man. The shortest period of compression that will ordinarily set up albuminuria is ten seconds.

### Brain Abscesses of Nasal Origin.

Donalies, in the *Arch. f. Ohrenh.*, reports the case of a boy of twelve who, as the result of a fall on to his forehead, developed a swelling above the root of the nose. Suffering from pain in the head, and with a hæmorrhagic nasal discharge which appeared to come from under the middle turbinated bones of both sides, he was admitted to hospital with a temperature of 102° F. The forehead swelling was opened and found to contain pus, which was also present in both frontal sinuses. A week later left-sided convulsions were noticed, and the right side of the brain was explored, but without result. On the left side, however, were found a small brain abscess and a larger subdural one, both of which were evacuated. The child subsequently made a satisfactory recovery.

### A Danger of the Trendelenberg Position.

A patient, aged 46, suffering from complete but reducible uterine prolapse, was operated upon in the Trendelenberg position and hystero-pexy with three sutures performed. The subsequent onset of intestinal obstruction necessitated further interference, and the mesentery was found twisted, with a volvulus of the ileum. Beneath the inferior border of the mesenteric loop were caught up two coils of small intestine, one of which was tightly nipped. Dr. H. Duret, who publishes the case in the *Journal de Science Médicale de Lille*, attributes the obstruction to the position of the patient at the time of operation. The throwing back of the viscera at an angle of 45° causes some amount of disturbance to their normal relations, especially in those who, like the patient, are the subjects of visceroptosis, and when, at the end of the operation, the horizontal position is resumed, the mesentery easily turns upon itself, and a volvulus is produced, which usually occurs from left to right owing to the relative fixation of the cæcum. The author, therefore, advises care in the use of the Trendelenberg position when dealing with cases of enteroptosis; the operating table should be moved into position slowly, and the abdomen should not be closed after operation until search has been made for twisted mesenteric loops and volvuli.

### Chlorinated Lime in Erysipelas.

In the *Berliner Klinische Wochenschrifte* the use of an ointment consisting of one part of chlorinated lime and nine parts of paraffin ointment is highly recommended by Binz for the treatment of erysipelas. After experience with fifteen cases of this nature to which he applied the ointment he finds that the temperature falls in three days, instead of nine as with ordinary methods, and no complications are likely to occur. He has treated cases of frostbite with the same ointment and found the results satisfactory. In a few cases the patient does not tolerate the ointment at full strength. In such cases it should be diluted to 5 per cent., and care be taken to prevent it coming into contact with any surfaces bare of skin. Sodium thiosulphate may be employed to remove the effects of chlorine if desired.

### Duodenal Ulcers in Children.

While duodenal ulcers are more rarely met with than gastric ulcers in children, the occurrence of either variety is less common in them than in adults. Both in the child and the adult a duodenal ulcer would seem to be the result of embolism and thrombosis, the portion of mucous membrane excluded in this way from the circulation being digested and destroyed. In both, again, the symptoms of duodenal ulcer are the same, but at times diagnosis is very difficult in the child. Comby and Kuttner, who have reported several cases in *les Archives de Médecine des Enfants*, believe that pyloric stenosis may at times occur in infants as the result of a reflex spasmodic contraction of the pylorus caused by a duodenal ulcer. The possibility of the existence of a similar cause must also be borne in mind when dealing with obscure anæmic conditions in young children. The existence of blood in the stools on microscopic examination will permit of a positive diagnosis being made. Children are best treated dietetically. In infants mother's milk should be given in small quantities at frequent intervals. For older children iced skim-milk in small quantities should be ordered. Alkaline medicines, such as magnesium carbonate, lime-water, Vichy or Carlsbad water and bismuth, give the best results. If there is hæmorrhage, ice should be applied to the epigastrium. Surgical intervention should only be resorted to when the diagnosis is certain, and then only if the hæmorrhages are frequent, and if medical treatment has been tried and found wanting.

### A New Vocation Dermatosis.

Dr. H. E. Alderson draws attention in the *California State Journal of Medicine* to a dermatitis resulting from the use of dry fulminate of mercury in a new process for the manufacture of blasting caps. Several workmen in the employ of a cap company of San Francisco developed a severe type of the disease, which appears after a very short exposure to the chemical and rapidly increases in severity. New papules may appear daily for some time after the onset of the inflammation. The skin would seem to be particularly susceptible to the irritating effects of the chemical when warm and perspiring. The dry fulminate is prepared by dissolving metallic mercury in nitric acid and precipitating with alcohol. One man, whose duty it was to prepare the drug, presented an acute erythemato-papular eruption involving the face, neck, post-aural regions, and the entire skin of both forearms, the hands only being spared, presumably on account of the thickness of the skin in those parts. The eyelids were acutely inflamed and there was a marked conjunctivitis. The eruption was most severe where the skin was thinnest. A pustular folliculitis was present on the bearded portion of the face. The attack had lasted for two weeks before the patient applied for treatment. Soothing applications cured the condition in a week, but it rapidly reappeared on the patient resuming the same employment. After experimenting with various salves as protective dressings, the best results were obtained with an ointment containing sodium carbonate ℥j and lanoline ℥ij smeared well over the exposed parts. The use of this salve has enabled the men to handle the fulminate with impunity, whereas before several had had to leave the company because of the dermatitis.

